# Remote cognitive training for older adults using tablets: A pilot trial

**DOI:** 10.1177/20552076261417771

**Published:** 2026-02-23

**Authors:** Liliana Mendes, Joana Oliveira, Marco Simões, Marta Pinto, Miguel Castelo-Branco

**Affiliations:** 1 Coimbra Institute for Biomedical Imaging and Translational Research/Institute for Nuclear Sciences Applied to Health, University of Coimbra, Coimbra, Portugal; 2 Faculty of Medicine (FMUC), 37830 University of Coimbra, Coimbra, Portugal; 3 Centre for Informatics and Systems/ Department of Informatics Engineering, 451611 University of Coimbra, Coimbra, Portugal; 4 Intellicare, Intelligent Sensing in Healthcare Lda, Coimbra, Portugal

**Keywords:** Cognitive training, older adults, tablet, assistive technology, cognitive impairment

## Abstract

**Background:**

Cognitive decline significantly affects the functional and intrinsic capacities of older adults, highlighting the need for effective interventions. Evidence suggests that mentally stimulating activities, particularly those supported by digital technologies, can promote cognitive health and quality of life in aging populations.

**Objective:**

This pilot trial examined the feasibility and preliminary effectiveness of *GameAAL*, a multidomain Cognitive Training programme delivered via tablet and television, in older adults with cognitive impairment or dementia.

**Methods:**

The intervention targeted key cognitive domains including attention, reaction time, memory, language, and executive functioning. Forty-one older adults (aged 60–93), living in nursing homes, participated in a 6-month programme. The tablet intervention group (*n* = 10) completed 30 sessions using a tablet device, while the TV intervention group (*n* = 31) completed nine sessions using a TV interface. All participants engaged with six serious games designed around cognitive tasks related to activities of daily living.

**Results:**

Pre- and post-intervention assessments included the Montreal Cognitive Assessment (MoCA) and the Hospital Anxiety and Depression Scale (HADS). The Tablet group showed a trend towards improved MoCA scores following the intervention, whereas the TV group did not show significant changes. At the post-intervention, the Tablet group demonstrated significantly better cognitive performance compared to the TV group (*p* = 0.044). No significant between-group differences were observed in HADS scores.

**Conclusion:**

The findings suggest that the *GameAAL* Cognitive Training programme may help improve cognitive function in older adults with cognitive impairment by combining computer-based exercises with ecologically valid tasks.

The rapid aging of the global population and its consequences are a growing concern. According to projections by the World Health Organization,^1^ the proportion of people aged over 60 is expected to nearly double between 2015 and 2050, reaching approximately 22% of the world’s population, or around two billion individuals. Coupled with increasing life expectancy, this trend highlights the rising prevalence of neurodegenerative dementias, particularly Alzheimer's disease and related disorders.^2^ The growing burden of dementia poses significant social and economic challenges for healthcare systems, care providers, and families, with notable disparities in access to care and resources among older adults.^3^ Beyond dementia, diabetes, falls, and depression are also major causes of disability in this population.^1^

Cognitive decline reduces the intrinsic capacity of older adults and is associated with higher risks of functional impairment and mortality.^4^ Although physiological and structural changes are inherent to aging, research indicates that regular participation in physical exercise and mentally stimulating activities, such as reading, puzzles, and social engagement, is linked to better cognitive performance, greater functional independence, improved emotional well-being, and reduced mortality risk.^5,6^ While healthy aging involves preserving functional capacity and adapting to psychological, physical, and social changes, age-related cognitive decline is often associated with reduced in activities of daily living and overall functional ability.^7–9^ Recent studies emphasize that advancements in cognitive interventions, particularly those supported by digital technologies, can enhance cognitive functioning^10^ and positively influence quality of life and well-being in older adults.^11^

## Cognitive training for the elderly delivered through information and communication technology

Cognitive interventions for older adults, with or without cognitive impairment, encompass seven main approaches: Compensatory Cognitive Training, Cognitive Stimulation, Enrichment, Cognitive Rehabilitation, Cognitive Training, and Cognitive Remediation and Brain Training.^12^ Although grounded in distinct theoretical models, these methods share the goal of preventing cognitive decline, sustaining cognitive abilities, or enhancing specific domains,^13^ and can be delivered via traditional or digital formats. In our previous review, we use the term computerized cognitive training (CCT) to refer to structured, goal-oriented cognitive exercises delivered through digital platforms. This construct encompasses interventions described in the literature ICT-based cognitive programmes, digital brain training tools, or technology-supported enrichment activities, provided they involve targeted practice of specific cognitive functions.

In this study, we employed a multidomain CCT programme targeting language, memory, attention, and executive functioning in older adults with cognitive impairment or dementia. The programme integrated guided practice, repetitive training, and individualized feedback to support skill acquisition and monitor progress.^10,14–22^ Evidence from recent meta-analyses indicates that technology-based interventions can yield improvements in global cognition, attention, processing speed, executive function, immediate recall, and working memory, with stronger effects in individuals with cognitive impairment and when programmes include structured tasks, professional supervision, and extended training schedules (exceeding 24 sessions of at least 30 min each).^23^ Collectively, findings suggest that multidomain, supervised, and adequately dosed cognitive programmes tend to produce the most robust effects.

Prior research using CCT in older adults with mild cognitive impairment (MCI) has reported gains in verbal learning and memory^24,25^ processing speed and attention,^26^ and working memory, functional outcomes, and subjective memory perception.^27^ Additional trials have shown improvements in attention, working memory, verbal fluency, and cognitive flexibility among individuals with MCI or mild dementia,^28^ as well as moderate gains in global cognition maintained over 6 months in older adults at risk of dementia.^29^ Meta-analytic evidence further supports enhancements in memory domains, particularly when training is supervised,^30^ and highlights the importance of frequency and duration for optimal effects.^31^ Despite promising results, long-term follow-up studies remain needed to determine sustainability and refine implementations for clinical settings.

Evidence from cognitively healthy older adults also supports structured CCT. The Advanced Cognitive Training for Independent and Vital Elderly (ACTIVE) trials remain the most influential in this field, examining multi-session programmes delivered to community-dwelling older adults over 5–6 weeks, typically comprising 10 training sessions focused on memory, processing speed, and reasoning.^32–37^ Some protocols included booster sessions.^33,35–37^ These interventions consistently produced domain-specific improvements, with reasoning and processing speed gains observed across cohorts^32,33^ and comparable benefits from memory-focused training.^34,35,37^ Effects were maintained for up to 5 years,^37^ and some outcomes, including preserved IADL (instrumental activities of daily living) functioning, remained detectable 10 years later.^36^ Booster training strengthened long-term maintenance, particularly for reasoning and processing speed.^35,36^ Additionally, long-term analyses linking ACTIVE data with mortality records indicated that higher baseline cognitive scores were associated with reduced mortality risk over a 20-year follow-up period, whereas the type of cognitive training received did not significantly influence survival outcomes.^38^ The subsequent study from Chen and colleagues seems to have found results in the same line.^39^ Interestingly, another ACTIVE follow-up study showed that participants enrolled in the cognitive training had a low risk for Alzheimer's disease and other related dementias associated to higher health care and education access indicators, over a 20-year follow-up period.^40^ Furthermore, the follow-up study of Zahodne et al.^41^ raised the attention to the role of perceived control. Although no moderation effects were found between intervention group and time to diagnosis, participants with greater levels of perceived control at baseline, evidenced lower dementias incidence.

Online-delivered programmes have yielded similar cognitive benefits. Gigler et al.^42^ reported enhanced working memory and processing speed following a ten-week, 17-session programme in adults with and without MCI. Similarly, Vermeij et al.^43^ observed improvements in digit and spatial span after a 5-week, 25-session intervention, with gains sustained at 3 months and modest enhancements in figural fluency.

Multidomain interventions, including CCT, have also demonstrated positive outcomes in dementia populations, with improvements reported in global cognition,^44–46^ physical functioning,^44^ mood,^46^ IADL performance, and reductions in anxiety and depressive symptoms.^44,46^

Tablet-based programmes represent another accessible delivery modality. Saluvich et al.^47^ found improvements in episodic memory, potential gains in visuospatial ability, and increased motivation and memory confidence in individuals with a-MCI after an eight-session intervention. Shamir et al.^48^ similarly reported reduced cognitive complaints following five group-based tablet sessions in older adults with MCI. Positive effects have also been observed in cognitively healthy older adults, including improved episodic memory, processing speed,^49,50^ and attentional control.^51^ Finally, Fong and colleagues^52^ also found favourable results with a 5-month CCT in a group with and without risk of MCI, prolonged even after a 3-month follow-up period.

Recent systematic reviews and meta-analyses reinforce the potential of ICT-based cognitive interventions for individuals with MCI or early dementia. Chae and Lee^53^ reported cognitive improvements, reduced depressive symptoms, and enhanced quality of life, particularly in programmes lasting at least 6 weeks, with sessions longer than 30 min and multidomain content. Jung et al.^54^ similarly observed statistically significant, albeit modest, cognitive gains with ICT-based interventions in MCI populations.

Although research on television-based Cognitive Training in older adults remains limited, findings from two randomized controlled trials (RCTs) are promising. Shatil et al.^55^ evaluated an interactive TV programme in 119 cognitively healthy older adults and found significant gains in working memory and executive function. Similarly, Nouchi et al.^56^ tested TV-based Cognitive Training games designed to enhance driving-related skills in 55 older drivers; over 6 weeks, adaptive training led to superior improvements in driving performance, processing speed, and inhibitory control, compared with non-adaptive games. These findings indicate that interactive TV platforms may provide an accessible and effective alternative for older adults, particularly those with limited computer access.

A summary of the main intervention characteristics, study populations, delivery modalities, and cognitive outcomes is provided in Table 1 to facilitate synthesis of the reviewed evidence.

**Table 1.** Summary of studies on cognitive training interventions, including interventions, study populations, delivery modality, and cognitive outcomes.

Author(s)/year	Interventions	Study populations	Delivery modality	Cognitive outcomes
Barnes et al.^24^	Auditory processing speed: 6-week programme comprising 30 sessions	47 older adults with MCI	CCT	Verbal learning and memory improvements in the intervention group; language and visuospatial measures favored control group
Belleville et al.^25^	Episodic memory: 8-week programme comprising nine sessions	28 with MCI and 17 cognitively healthy older adults	CCT	Significant gains in delayed list recall and face–name association; improvements also observed in subjective memory and well-being; no change in the non-intervention control group
Zelinski et al.^26^	Processing speed and auditory processing: 8–10 weeks programme comprising 40 sessions	487 older adults without significant cognitive impairment	CCT	Significant improvement in memory and attention, processing speed; effects maintained but reduced at 3-month follow-up
Hyer et al.^27^	Working memory: 5–7 weeks programme comprising 25 total sessions	68 older adults with MCI	CCT	Both groups improved, but Cogmed produced greater working memory gains, higher subjective memory ratings, and better functional adjustment, with participants, especially in the Cogmed group, reporting high satisfaction
Diaz Baquero et al.^28^	Memory and executive functioning: 2–3 weekly sessions for 4 months	43 older adults with MCI or mild dementia	CCT	Improved attention, working memory, phonological verbal fluency, and cognitive flexibility
Bahar-Fuchs et al.^29^	Global cognition: 8 weeks	84 older adults at higher dementia risk	CCT	Moderate improvements in global cognition; gains maintained at 6-month follow-up
Wolinsky et al.^32^	Reasoning, processing speed, or memory: 6 weeks programme comprising 10 sessions	1534 older adults	CCT	Reasoning and processing speed training led to medium-sized 5-year gains in internal locus of control, with no benefits from memory training or on other locus of control measures
Ball et al.^33^	Processing speed: 10 initial sessions plus up to 8 booster sessions	2802 older adults	CCT	Processing speed gains lasted 5 years, with booster sessions boosting effects
Jones et al.^34^	Memory, reasoning and processing speed: 5–6 weeks programme comprising 10 sessions	1659 older adults	CCT	Memory gains were fully maintained over 5 years; reasoning and speed gains were partially lost but still exceeded control group performance. At follow-up, performance differences corresponded to approximately 6 years (memory), 4 years (reasoning), and 8 years (speed) of age-related decline. Only reasoning training showed signs of slowing normative cognitive decline
Sisco et al.^35^	Memory, reasoning, and processing speed: 5–6 weeks programme comprising 10 sessions	1912 older adults	CCT	Memory training improved verbatim recall, with booster sessions enhancing the effect. However, the benefits did not persist after one year without continued practice
Rebok et al.^36^	Memory, reasoning and processing speed: 5–6 weeks programme comprising 10 sessions	2832 older adults	CCT	After 10 years, around 60% of trained participants maintained their baseline IADL function, compared to 50% of controls. Long-term improvements in cognitive abilities were sustained for reasoning and processing speed, but not for memory. Booster sessions provided additional lasting benefits for reasoning and speed performance
Rebok et al.^37^	Memory, inductive reasoning and processing speed: 10 initial sessions plus 4 booster sessions	2802 older adults	CCT	Memory training led to improved memory performance for up to 5 years. However, booster sessions and adherence did not significantly enhance this effect. Age influenced memory decline over time, while higher education
Rebok et al.^38^	Memory, reasoning, or processing speed: 5–6 weeks programme comprising 10 sessions	2802 older adults	CCT	Higher baseline cognitive performance predicted lower mortality risk over 20 years; however, no significant effect of training type (memory, reasoning, or speed) on mortality risk was observed
Chen et al.^39^	Memory, reasoning, processing speed	2802 older adults	CCT	Higher baseline cognition, slower decline, and stronger retest effects were associated with lower mortality; cognitive training itself showed no significant effect on cognitive trajectories or mortality
Rebok et al.^40^	Cognitive training programmes delivered within the ACTIVE trial	1605 older adults	CCT	Improved neighborhood and environmental conditions were associated with a reduced risk of Alzheimer's disease and related dementias. Cognitive training appeared more effective for participants with better access to health care and educational resources, though the overall influence of social determinants of health on training outcomes was more limited than expected
Zahodne et al.^41^	Memory, reasoning, processing speed	2021 older adults	CCT	Higher perceived control at baseline was linked to a lower risk of developing Alzheimer's disease and related dementias. Perceived control did not alter how the different cognitive training interventions affected ADRD incidence
Gigler et al.^42^	General cognitive function: 10-week programme comprising 17 sessions	7 with MCI and 11 cognitively healthy older adults	Online-BCP	Significant gains in working memory and processing speed
Vermeij et al.^43^	Working memory: 5-week programme comprising 25 sessions	23 healthy older adults and 18 with amnestic MCI	Online-BCP	Both groups improved on digit span and spatial span, with gains maintained at 3-month follow-up. Healthy participants, and to a lesser degree those with MCI, also showed improvement in figural fluency. No evidence of broader transfer to other cognitive domains
Chae & Lee^44^	Multidomain function: 8-week programme comprising 40 sessions	60 older adults with dementia	Online-BCP including Tablet	Significant improvements in cognitive function, physical ability, and nutritional status; reduction in depression
Chau et al.^45^	Upper limb/finger movement, arithmetic, and verbal fluency: 12-week programme comprising 24 sessions	76 older adults	Online-BCP including Tablet	Feasible and well-accepted, with 82% reporting satisfaction. The intervention group demonstrated medium-to-large within-group gains in subjective memory and cognitive function from midintervention to follow-up, and small-to-medium between-group effects compared to controls
Jung et al.^46^	Cognition, mood, and IADL: 4-week internet-based and 4-week in-person comprising 32 sessions	42 older adults with mild to moderate Alzheimer's disease	Online-BCP	Both formats significantly improved cognition, depression, anxiety, and IADL compared with controls (in-person). Cognitive and depression outcomes were similar between formats, while in-person training showed greater benefits for anxiety and IADL
Savulich et al.^47^	Episodic memory and visuospatial: 4-week programme comprising eight sessions	42 older adults with a-MCI	Tablet device	Cognitive training significantly improved episodic memory and has the potential to enhance visuospatial abilities in individuals with a-MCI, with participants showing increased motivation, confidence, and perceived memory ability over time
Shamir et al.^48^	General cognitive function: 5-week programme involving weekly group sessions	14 older adults with MCI	Tablet device	Reduced cognitive complaints
Chan et al.^49^	Processing speed, mental control, episodic memory, visuospatial processing: 3 months (15 h/week)	54 older adults	Tablet device	Compared to the control groups, participants in the tablet training group showed improvements in episodic memory and processing speed, with no significant changes observed in mental control or visuospatial skills
Vaportzis et al.^50^	Verbal comprehension, perceptual processing, working memory, processing speed: 10-week programme comprising 10 sessions	43 healthy older adults (22 intervention group; 21 control group)	Tablet device	Tablet-based training improved processing speed compared to a no-contact control group. Findings suggest that cognitively engaging activities, such as learning new skills, may enhance processing speed earlier than other cognitive domains
Binder et al.^51^	Executive functions, attentional control, working memory and speed: 10-week programme comprising 50 sessions	84 healthy older adults	Tablet device	Participants in the multi-domain training group showed greater improvement in attentional control compared to those in single-domain training groups. Those with lower initial cognitive performance benefited the most from the training. These gains were maintained at a 6-month follow-up
Fong et al.^52^	Memory, eye–hand coordination, attention, calculation, and judgement	134 older adults, with and without risk of MCI	Tablet device	Both groups showed significant cognitive improvements after training, maintained at 3-month follow-up. The MCI-risk group had larger gains in overall cognitive functioning immediately post-intervention, particularly in attention and eye–hand coordination, while the non-MCI group improved more in memory and judgment. Results support mobile-based CCT as an effective and practical intervention for cognitive enhancement in older adults
Shatil et al.^55^	Working memory, executive control and analogical reasoning: 8-week programme comprising 24 sessions	119 healthy older adults	TV-BCT	Significant improvements in working memory and executive function in the training group; no improvements in the control group (engaged in three non-cognitive applications)
Nouchi et al.^56^	Processing speed, dual attention, and speed prediction: 6-week programme comprising 30 sessions	55 healthy older adults. (27 intervention group; 28 control group)	TV-BCT	Intervention group improved driving skills, processing speed, and inhibition; no improvements in the control group (non-adaptive training, with game difficulty remaining constant throughout the intervention)

Note: MCI: Mild Cognitive Impairment; CCT: Computerized Cognitive Training; IADL: Instrumental Activities of Daily Living; Online-BCP: Online-Based Cognitive Programmes; a-MCI: Amnestic Mild Cognitive Impairment; TV-BCT: TV-Based Cognitive Training.

A pilot trial was conducted to evaluate the feasibility and preliminary effectiveness of the ‘GameAAL—Gamification Supporting Active and Assisted Living’ programme, a tablet and TV-Based Cognitive Training intervention targeting attention, reaction time, memory, language and executive functioning, which incorporate ecological tasks to promote the transfer to participants’ everyday life.

To date, studies are still scarce in examining the application of two simultaneous types of intervention, tablet and TV-Based Cognitive Training, in older adults with cognitive impairment or dementia, and reflect their performance in parallel based on the same interface, from the *Neurohab* platform,^57^ previously tested in a pilot study with a population of heterogeneous cognitive impairment and dementia, using the tablet modality.^58^ This software platform provides several features, including real-time feedback and personalized learning (for more details see^57^). This underscore the importance of this pilot study for assessing feasibility, adherence, user-friendliness, the level of acceptance and attendance in this population and, ultimately, the potential cognitive benefits from these paralleled interventions (tablet and TV modalities), before progressing to a definitive trial, serving as a final proof of concept. We hypothesized that participation in this intervention would result in improvements in overall cognitive functioning.

## Method

### Participants

A convenience sample was recruited through contact with nursing homes in the Coimbra district. Three institutions were selected, whose residents presented varying levels of cognitive impairment. The study was approved by the Ethics Committee of the Faculty of Medicine of the University of Coimbra (CE-077/2018), and written informed consent was obtained from all participants or their legal representatives. All procedures were conducted in accordance with the Declaration of Helsinki.

**Figure 1.**
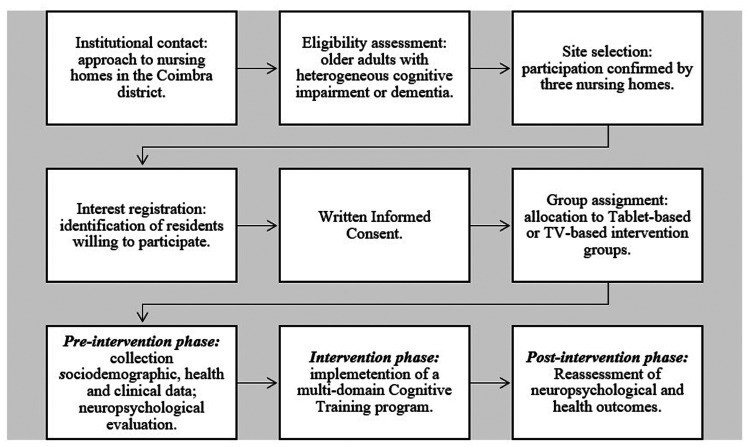
Study procedure.

Eligibility criteria were verified, and informed consent was obtained from all participants or their legal representatives (see Figure 1). This pilot study was initially designed as a parallel-group RCT with a 1:1 allocation ratio. However, practical constraints during implementation meant that a true randomization procedure could not be applied. Consequently, participants were allocated manually in the order of their enrollment and according to their availability for scheduled sessions. The allocation was carried out by administrative staff who were not involved in participant assessment or data handling, and no demographic, clinical, or cognitive information was used in the assignment process. Participants were allocated based on predefined inclusion and exclusion criteria, namely: *Inclusion criteria:* (1) age 60 years or older; (2) ability to remain on task; (3) evidence of cognitive impairment or dementia; (4) no diagnosis of a psychopathological disorder; and (5) motivation to participate in the Cognitive Training programme; *Exclusion criteria*: (1) severe neurological disorders; (2) alcohol and/or drugs abuse; and (3) reduced insight. The final sample comprised 41 participants.

### Outcome measures

The evaluation protocol included two standardized assessment tools designed to measure global cognitive functioning and symptoms of anxiety and depression. An anamnesis interview was also conducted to collect sociodemographic and clinical data. The selection of instruments was based on several criteria: representativeness, relevance of the constructs measured, cultural and linguistic adaptation for the Portuguese population, empirical validation, and the availability of normative data. Together, these measures enabled a comprehensive characterization of the sample in sociodemographic and clinical terms, and provided an objective evaluation of the participants’ neuropsychological functioning. This approach allowed for the quantification of both impairments and preserved abilities across cognitive domains, distinguishing normal aging processes from potential neuropsychopathological conditions.

1. Sociodemographic Questionnaire: as part of the anamnesis, personal information, health status, and clinical history were collected to characterize the sample.
2. Hospital Anxiety and Depression Scale (HADS^59,60^: symptoms of anxiety and depression were assessed using the validated Portuguese version of the HADS. This self-report instrument is widely used in clinical and research settings to screen for emotional distress. Given that the sample consisted primarily of older adults, the HADS items were administered verbally by trained the assessors to ensure participants full comprehension, particularly among those with potential literacy or visual difficulties. The original authors note that individuals with limited literacy may experience embarrassment when completing self-report questionnaires ^61^; this consideration informed the decision to adopt a standardized verbal administration procedure in the present study. To minimize interviewer bias, all assessors followed a standardized administration protocol, reading each item verbatim in a neutral pace and tone, without paraphrasing, providing explanations, or emphasizing any response options.
3. Montreal Cognitive Assessment (MoCA^62,63^: the MoCA is a brief cognitive screening tool used to differentiate between age-related cognitive changes and those associated with pathological conditions. It has been extensively validated in the Portuguese population. The MoCA evaluates eight cognitive domains: executive functions, visuospatial abilities, short-term memory, language, attention, concentration and working memory, and temporal and spatial orientation.^54–68^ The maximum *score* is 30 points, with normative data stratified by age and educational level.^68^

Outcome assessors were not blinded to group allocation because they also provided support during the intervention sessions. To minimize possible assessment bias, all evaluations were conducted using standardized administration procedures.

### Procedure

**Figure 2.**
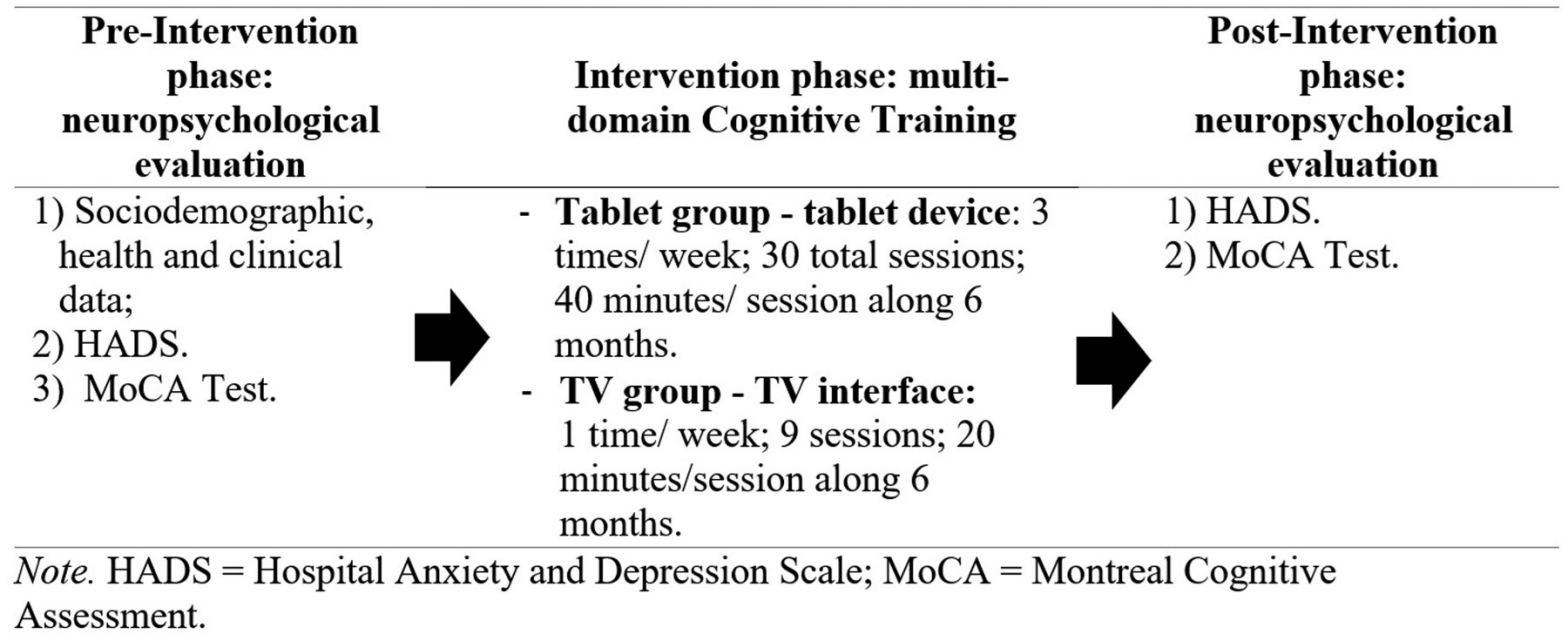
Study phases.

This study comprised three phases: pre-intervention, intervention, and post-intervention, as summarized in Figure 2.

#### Pre-intervention

The first phase consisted of a single neuropsychological assessment session conducted individually with each participant. These evaluations were administered on-site by clinical psychologists experienced in neuropsychological assessment. In line with the established protocol, the purpose of this session was to characterize participants’ baseline cognitive and emotional functioning, enabling later comparison with post-intervention outcomes. The duration of each session varied depending on individual performance, but on average the full protocol required approximately 30 min to complete.

#### Intervention phase

The intervention sessions consisted of multidomain Cognitive Training activities (see Table 2). Neuropsychologists were responsible for designing the exercises and conducting the sessions, including those delivered via TV. The programme was developed to stimulate several cognitive domains: some tasks targeted a specific cognitive function, while others required the simultaneous engagement of multiple low-level processes. Training focused on four core domains: (i) memory; (ii) attention; (iii) language; and (iv) executive functions (see Table 2).

**Table 2.** Cognitive functions trained and description of the serious games.

Serious games	Main cognitive functions trained			
	Memory	Attention	Language	Executive functions
Puzzle				
Maze				
Quiz				
Reaction games				
Whack-a-mole				
Pairs				
Word, symbol, and number search				

*Note:* The shaded cells means that the specific serious game is responsible to train the correspondent cognitive function.

To maintain an appropriate level of challenge and prevent boredom, frustration, or demotivation, the exercises were adapted to each participant’s cognitive abilities by adjusting task parameters (e.g. speed, number of stimuli). This adaptive feature enables personalized learning tailored to individual needs.

The content of the activities was linked to participants’ interests, incorporating elements such as crosswords, common Portuguese proverbs, and tasks connected to daily life. The application design also integrated features highlighted in the literature as essential for older adults: (i) sensitivity to slower response times and reduced sensory acuity (e.g. large fonts, large buttons, and clear stimuli); (ii) multiple levels of difficulty and categories; (iii) real-time performance feedback; (iv) visual and auditory reinforcement; (v) participant awareness of the training’s relevance; and (vi) the absence of time limits.^69–75^ These features were embedded in the training interface used in this study.

To promote the transfer to everyday life, several exercises were ecological in nature, requiring participants to solve tasks resembling real-life situations. The Cognitive Training included a variety of serious games designed to stimulate different cognitive processes: (i) *Puzzle*, (ii) *Maze*, (iii) *Quiz*, (iv) *Reaction Games*, (v) *Whack-a-Mole*, (vi) *Pairs*, and (vii) *Word, Symbol and Number Search*, as detailed in Appendix 1. Some of these tasks had been described previously in a study by Simões et al.^58^

The tablet-based intervention sessions were held three times per week using tablet devices. The technology was selected for its high interactivity and motivational potential, which facilitate adaptation and engagement among older adults. Its portability also allows for greater flexibility, as tablets are not dependent on external equipment or fixed locations. Sessions were conducted within each nursing home, lasted approximately 40 min, and extended over 6 months, totaling 30 sessions per participant, consistent with the findings of Chen et al.^23^ All sessions were conducted by neuropsychologists, whose primary role was to support participants during the activities, monitor performance, and provide feedback. They also offered motivational support and ensured participants remained engaged throughout the tasks. Attendance, completion rates, and in-app activity data were automatically recorded by the platform, allowing objective monitoring of adherence and engagement over the course of the intervention.

The TV group participated in weekly sessions of the multidomain Cognitive Training programme, following the same principles as the Tablet group. All sessions were held in the three nursing homes, lasted approximately 20 min, and spanned six months, totaling nine sessions per participants. The sessions were delivered via a Smart TV using an Android-based interface from the *Neurohab* platform.^57^ The system was organized around three core modules: (1) tasks simulating activities of daily living, (2) monitoring of health parameters, and (3) neurocognitive training delivered exclusively through serious games. The Cognitive Training module included the same games used in the Tablet group, also used in the intervention with the Tablet group: (i) *Puzzle*, (ii) *Maze*, (iii) *Quiz*, (iv) *Reaction Games*, (v) *Whack-a-Mole*, (vi) *Pairs*, and (vii) *Word, Symbol and Number Search*.

The modules operate in an interconnected and interdependent manner, integrating data collected from biosensors and environmental sensors (e.g. motion detectors), to programme the training sessions and select the targeted cognitive domains. All sessions were supervised by biomedical professionals with specialized training in the area. Their primary role was to support participants during the activities, oversee task execution, provide feedback, and offer motivational reinforcement when required.

Data collected through the interface were processed by an external unit installed remotely, enabling continuous monitoring of task performance. The system generated activity patterns, identified deviations, and allowed for the personalized planning and adjustment of each session. In addition, algorithms incorporated into the system were capable of generating real-time alerts within the local unit. Session attendance, duration, and completion were automatically logged by the platform, allowing objective monitoring of adherence and engagement throughout the intervention.

To further enhance engagement, the platform was designed to promote positive reinforcement in collaboration with caregivers and institutional staff. Participants’ achievements and progress were rewarded with meaningful and enjoyable experiences, including cultural and leisure activities tailored to their preferences.

#### Post-intervention

All participants completed the final phase, which consisted of a post-intervention neuropsychological assessment session. This session followed the same structure and procedures as the pre-intervention phase, ensuring consistency in data collection.

The purpose of this phase was to determine magnitude of the intervention’s effects through objective outcome measures, allowing direct comparison with the participants’ pre-intervention performance.

### Statistical analysis

All statistical analyses were conducted using IBM SPSS, Version 23. As some variables did not meet the normality assumption (Shapiro–Wilk test), both parametric and non-parametric tests were initially computed. The results were consistent across methods; therefore, for clarity and interpretability, only parametric test results are reported in the main text. This decision is supported by methodological recommendations,^76^ which emphasize the robustness of parametric tests and the interpretability of mean-based outcomes.

MoCA scores were used to assess cognitive functioning at both pre- and post-intervention timepoints. Means and standard deviations were calculated, and score changes were examined by comparing timepoint 2 (post-intervention) with timepoint 1 (pre-intervention) within each intervention group using paired-samples *t* tests. Ninety-five percent confidence intervals were computed for the mean difference, and Cohen's *d* was calculated as the effect size.

To evaluate group differences in MoCA scores at both timepoints, independent-samples *t* tests were conducted to compare the Tablet and TV groups at pre- and post-intervention. Hedges’ *g* was reported as the effect size to account for unequal sample sizes. When appropriate, a mixed-design ANOVA was performed to examine the interaction between timepoint and group, with eta squared η^2^ reported as the corresponding effect size.

Pearson correlation coefficients was computed to explore the relationship between MoCA scores and HADS depression and anxiety scores. In addition, independent-samples *t* test was used to compare the Tablet and TV groups on their HADS depression and anxiety scores.

## Results

### Recruitment of participants

**Figure 3.**
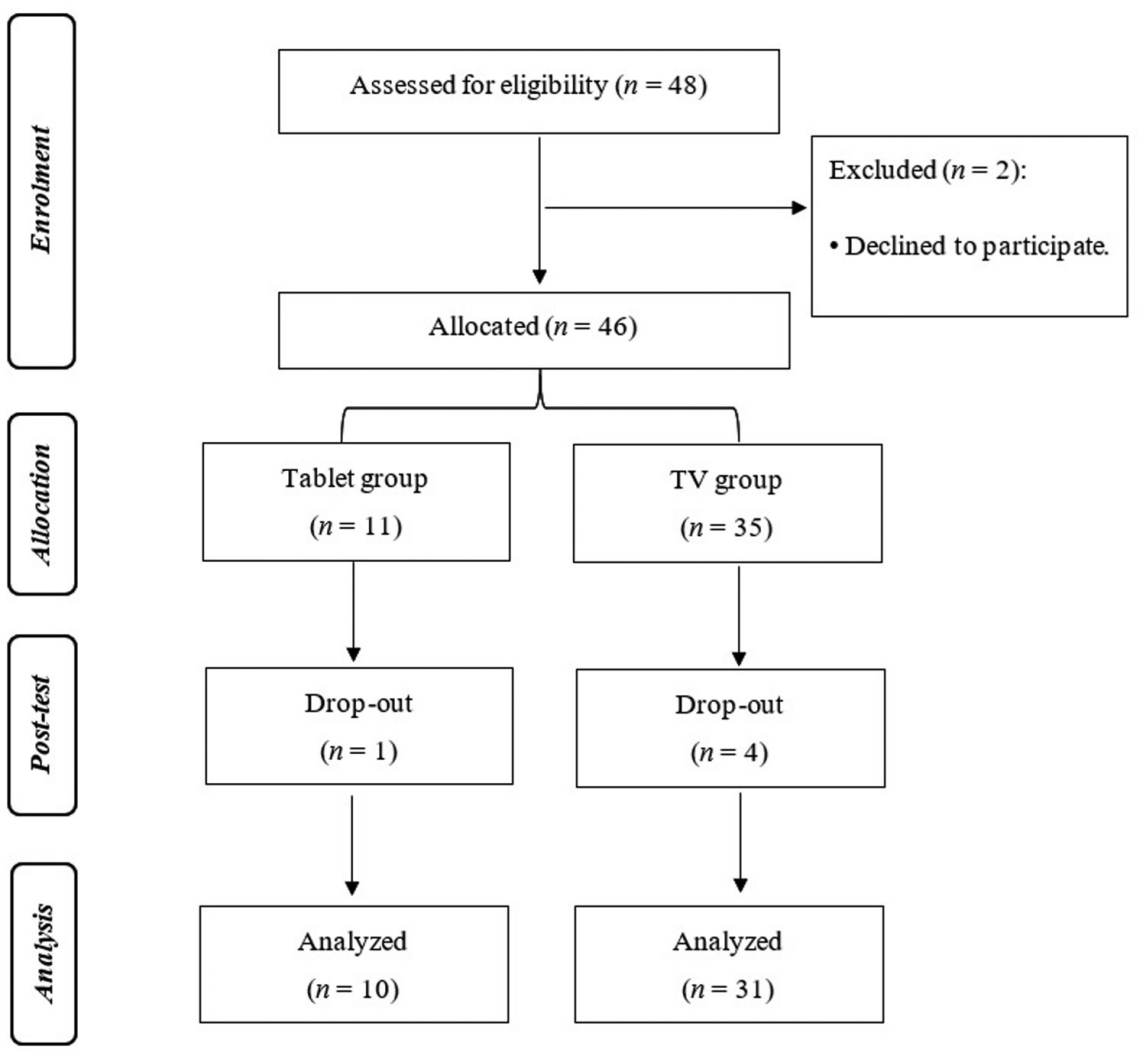
Flowchart of the non-randomized pilot trial.

A total of 48 older adults, who expressed clear interest in participating, were initially recruited to take part in a Cognitive Training programme delivered via tablet and TV interfaces. As this was a pilot study, the sample size was determined by practical considerations, including available resources, timeframe, and recruitment feasibility across sites. The primary aim was to evaluate the feasibility and acceptability of the study procedures rather than to test efficacy. During the intervention phase, five participants dropped out: three withdrew voluntarily, and two passed away. The final sample consisted of 41 participants (10 males and 31 females), aged between 60 and 93 years. Regarding educational background, six participants lacked reading and writing skills but had attended elementary school; 17 had not completed basic education; another 17 completed the first cycle of basic education; and one participant completed the second cycle. The sampling flowchart is presented in Figure 3.

### Intervention effects

Pre- (T1) and post-intervention (T2) assessment using the Montreal Cognitive Assessment (MoCA), along with the mean differences between timepoints, are reported in Tables 3 and 4 for the Tablet and TV groups, respectively.

**Table 3.** Differences between MoCA pre- (T1) and post-intervention (T2) results for the Tablet group (*n* = 10).

MoCA scores	Tablet T1	Tablet T2	T2–T1	*p*	*d*
	*M (±SD)*	*M (±SD)*	*M* (95% CI)		
MoCA_Visuospatial/Executive	1.60 (**±**1.26)	1.90 (**±**1.45)	0.30 (−0.53, 1.13)	0.434	0.24
MoCA_Naming	1.20 (**±**1.03)	1.30 (**±**1.06)	0.10 (−0.43, 0.63)	0.678	0.10
MoCA_Attention	2.70 (**±**1.89)	3.30 (**±**2.06)	0.60 (−0.30, 1.50)	0.168	0.32
MoCA_Language	0.80 (**±**1.23)	1.40 (**±**0.84)	0.60 (0.00, 1.20)	0.051	0.49
MoCA_Abstraction	0.40 (**±**0.84)	0.60 (**±**0.70)	0.20 (−0.25, 0.65)	0.343	0.24
MoCA_Delayed Recall	0.10 (**±**0.32)	0.20 (**±**0.42)	0.10 (−0.13, 0.33)	0.343	0.31
MoCA_Orientation	4.40 (**±**1.96)	4.70 (**±**1.64)	0.30 (−1.01, 1.61)	0.616	0.15
MoCA_Total	**11.20** **(± 6.68)**	**13.40** **(± 6.98)**	**2.20** **(−0.25, 4.65)**	**0.073**	**0.33**

Note: M: mean; SD: standard deviation; CI: confidence interval; *d*: effect size.

**Table 4.** Differences between MoCA pre- (T1) and post-intervention (T2) results for the TV group (*n* = 31).

MoCA scores	TV T1	TV T2	T2–T1	*p*	*d*
	*M (±SD)*	*M (±SD)*	*M* (95% CI)		
MoCA_Visuospatial/Executive	0.65 (**±**0.75)	1.13 (**±**0.56)	0.48 (0.25, 0.71)	<.001*	0.64
MoCA_Naming	0.94 (**±**0.85)	1.29 (**±**1.07)	0.35 (−0.01, 0.72)	.054	0.41
MoCA_Attention	1.58 (**±**1.43)	1.26 (**±**1.24)	−0.32 (−0.72, 0.07)	.106	−0.22
MoCA_Language	0.52 (**±**0.72)	0.26 (**±**0.51)	−0.26 (−0.47, −0.05)	.018*	−0.36
MoCA_Abstraction	0.23 (**±**0.50)	0.03 (**±**0.18)	−0.19 (−0.39, 0.01)	.056	−0.38
MoCA_Delayed Recall	0.16 (**±**0.58)	0.23 (**±**0.67)	0.06 (−0.17, 0.30)	.572	0.10
MoCA_Orientation	4.29 (**±**1.64)	3.94 (**±**1.73)	−0.35 (−0.95, 0.24)	.233	−0.21
MoCA_Total	**8.36** **(± 3.87)**	**8.13** **(± 3.84)**	**−0.23** **(−1.15, 0.70)**	**.622**	**−0.06**

Note: M: mean; SD: standard deviation; CI: confidence interval; * Significant results (*p* < 0.05); *d*: effect size.

Following the tablet-based intervention, improvements were noted across all MoCA subdomains, particularly in *language* and *attention*. However, these changes did not reach statistical significance (all *p* > 0.05).

In contrast, participants in the TV-based intervention group demonstrated negative variations in the *attention, language, abstraction* and *orientation* subdomains, resulting in an overall decline in the MoCA total scores. Statistically significant pre-post differences were detected in two domains: *visuospatial/executive*, which improved post-intervention (*p* < 0.001), and *language*, which demonstrated a decline (*p* = 0.018) in performance following the intervention.

**Figure 4.**
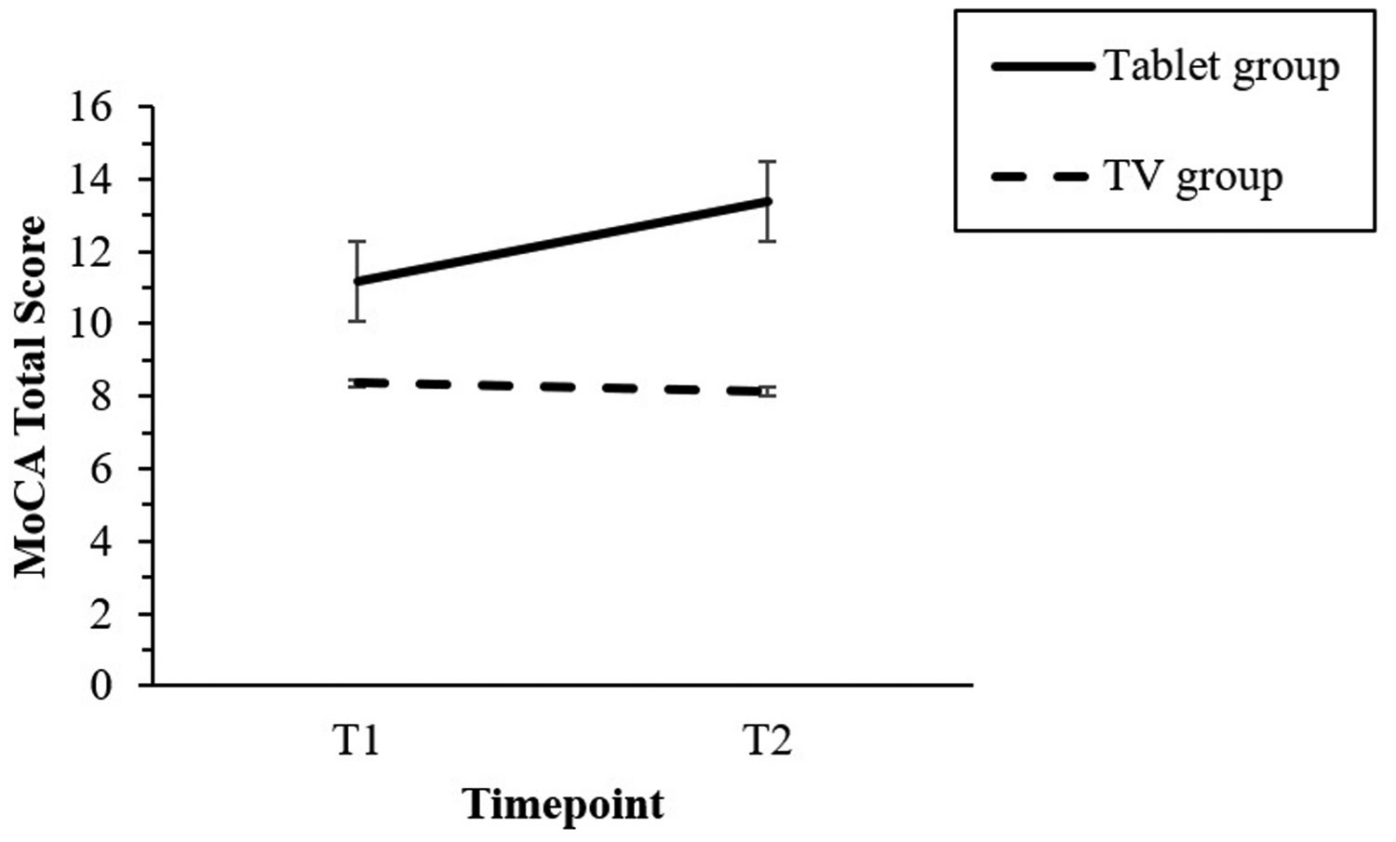
Timepoint by group interaction effect on MoCA total score. Only Tablet group revealed a superior performance after the intervention, comparing to the baseline point.

When comparing MoCA total scores between groups, significant differences emerged only at the post-intervention assessment (*p* = 0.044, *g* = 1.11), with the Tablet group outperforming the TV group, suggesting a positive effect of the tablet-based intervention. No significant group differences were found at the baseline (*p* = 0.102, *g* = 0.61). The effect of the timepoint × group interaction was statistically significant, *F*(1, 39) = 5.84, *p* = 0.020, η^2^ = 0.13, indicating that MoCA total scores improved from T1 to T2 exclusively in the Tablet group (see Figure 4).

Anxiety and depression levels, assessed with the HADS at both timepoint, were examined to ensure that no significant variations could have confounded the observed cognitive outcomes. No significant differences were found between the Tablet and TV groups for anxiety (*p* = 0.420, *g* = 0.30 and *p* = 0.991, *g* = 0.00, at pre- and post-intervention, respectively) or depression (*p* = 0.131, *g* = 0.56 and *p* = 0.344, *g* = 0.35, at pre- and post-intervention, respectively), thus minimizing the likelihood of their influence on MoCA scores improvements. Additionally, no significant correlations were detected between anxiety or depression scores and MoCA scores, at both timepoints (all *r* < 0.22, *p* > 0.05).

## Discussion

The primary aim of this study was to evaluate the effectiveness of the *GameAAL* Cognitive Training programme, delivered via tablet, in strengthening multiple cognitive domains, including attention, reaction time, memory, language, and executive functioning, compared with a TV-Based Cognitive Training format.

Following the intervention, participants in the Tablet group demonstrated improvements across all assessed cognitive domains, with the largest gains in language and attention. Although these improvements did not reach statistically significance, the direction of these effects aligns with findings reported by Shamir et al.^48^ By contrast, the TV group exhibited negative variations between pre- and the post-test MoCA scores in several domains, excluding naming, delayed recall, and visuospatial abilities, with the latter showing statistical significance. The only significant improvement in this group was observed in the visuospatial/executive domain between timepoints; while language performance significantly declines. These results are consistent with those of Shatil et al.,^55^ who also observed significant improvements in executive functioning following TV-Based Cognitive Training. Importantly, when comparing groups, the Tablet condition yielded statistically significant MoCA total scores at post-intervention (*p* = 0.044), suggesting a superior overall effect of the tablet-based intervention.

Several factors may account for these differences. First, the training intensity varied substantially: the Tablet group participated in three 40-min sessions per week, whereas the TV group engaged in a single 20-min session weekly. Greater exposure may have amplified learning opportunities for the Tablet group. Second, motivation and prior familiarity with technology could have influenced outcomes. Many participants in the Tablet group already engaged in cognitively stimulating activities (e.g. crosswords and puzzles) and were comfortable with digital devices. Their enthusiasm for tablet use was evident, and the programme’s individualized features, tailoring tasks to each participant’s skill level and preferences, likely further reinforced engagement. Direct interaction with a therapist, providing immediate feedback and motivational support, was also have enhanced adherence and performance. For a broad comprehension about factors related to compliance with tablet CCT programmes, see this study.^77^

Nevertheless, caution is warranted in interpreting these findings. Different training intensity, the participants’ baseline cognitive engagement, the differences in motivation levels, the individualized support provided by the specialists (including the difference in the facilitators’ expertise) and, consequently, the possible Hawthorne effect due to their presence, are some relevant aspects to consider not only as an explanation for the observed superior performance in the Tablet group, but also as a possible confounder to those results. These considerations highlight the need for future studies to systematically control for such variables when comparing different modes of delivery.

Despite these limitations, our findings suggest that sustained and tailored cognitive training may support engagement and functional outcomes in older adults, although further research is needed. We could anticipate that interventions sustained over time and adjusted to individual needs contribute to maintain the benefits, as suggested by previous authors^78,79^ and further corroborated by evidence from the ACTIVE trials.

The present study is not without limitations. The small sample size, from only three nursing homes within a single district, may have reduced statistical power and limited generalizability of the findings. Some caution is needed, as this population may not fully represent older adults in broader community settings. Another limitation concerns group allocation, which was performed manually rather than through true randomization, potentially introducing selection bias and reducing internal validity. Future research should replicate these findings in larger, blinded RCTs to strengthen evidence and support these findings on a broader scale. The restricted range of outcome measures also represents a limitation. Although the MoCA and HADS are valid and reliable instruments, their focus on global cognition and mood may have overlooked changes in functional performance or quality of life. Including standardized ADL/IADL or other ecologically oriented measures in future studies would provide a more comprehensive understanding of intervention effects. Furthermore, outcome assessors were not blinded to group allocation, which may have introduced assessment bias despite adherence to standardized administration procedures. In addition, the sample excluded healthy older adults and individuals with different dementia profiles, limiting the scope of conclusions. Finally, methodological differences between the two interventions formats (Tablet and TV groups), including variations in session frequency, intensity (e.g., the Tablet group received a more intensive intervention), and engagement, constraint the validity of direct comparisons. Advanced approaches such as meta-regression analyses could provide valuable information, for example, which populations derive the greatest benefit (e.g. people with dementia vs. healthy older adults), which type of Cognitive Training is most effective (e.g. multidomain vs. single-domain), and whether improvements generalize to daily functioning.

## Conclusion

In summary, findings from this pilot study suggest that the *GameAAL* Cognitive Training programme, delivered via tablet, may enhance cognitive functioning in older adults with cognitive impairment or dementia. The platform is relatively low-cost, widely accessible, and potentially scalable, offering promise not only as a preventive tool against age-related cognitive decline in healthy populations but also as a supportive intervention to preserve cognitive abilities in those already experiencing cognitive deterioration.

## Supplemental Material

sj-pdf-1-dhj-10.1177_20552076261417771 - Supplemental material for Remote cognitive training for older adults using tablets: A pilot trial Supplemental material, sj-pdf-1-dhj-10.1177_20552076261417771 for Remote cognitive training for older adults using tablets: A pilot trial by Liliana Mendes, Joana Oliveira, Marco Simões, Marta Pinto and Miguel Castelo-Branco in DIGITAL HEALTH

sj-doc-2-dhj-10.1177_20552076261417771 - Supplemental material for Remote cognitive training for older adults using tablets: A pilot trial Supplemental material, sj-doc-2-dhj-10.1177_20552076261417771 for Remote cognitive training for older adults using tablets: A pilot trial by Liliana Mendes, Joana Oliveira, Marco Simões, Marta Pinto and Miguel Castelo-Branco in DIGITAL HEALTH
