# Supplementary material for: Remote cognitive training for older adults using tablets: A pilot trial
Source: Digit Health. 2026 Feb 23;12:20552076261417771. doi: 10.1177/20552076261417771 (PMC12929873; doi:10.1177/20552076261417771)

Appendix 1

Cognitive Training protocol and description of serious games.

Serious Games description	Illustrative picture
**Puzzle**<br>This game requires problem-solving skills and visuospatial integration, thereby helping to train sustained attention. It consists of a set of puzzle pieces that must be assembled to reproduce an image matching a given stimulus. The user can select from various thematic categories for the game: Animals, Flowers, Fruit, Architecture, Tourism, Vehicles and Random theme.	**a) Screenshot of the Puzzle Game**<br>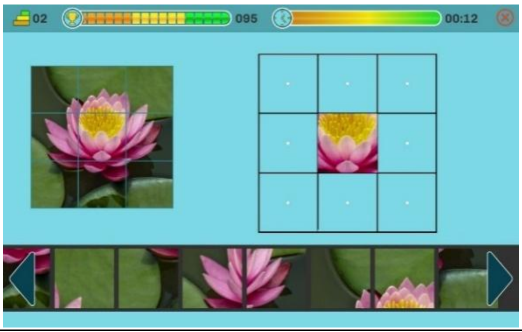
**Maze**<br>Designed to train problem-solving skills, the Maze game challenges the user to "drive" a car by guiding it with their finger along the correct path (also helping to develop motor skills), while avoiding crossing lines or obstacles. To successfully complete the task, the user must be able to plan their route in advance and employ abstract thinking and reasoning skills to navigate through the maze. The game concludes successfully when the vehicle reaches its destination.	**b) Screenshot of the Maze Game**<br>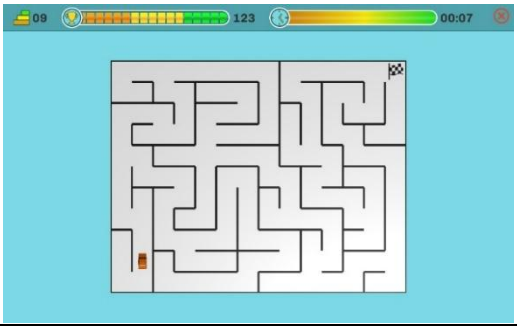
**Quiz**<br><br>**1) Money** – in this task, participants view a stimulus (coins or banknotes of various amounts) and respond to the question "What is the amount in the image?" by selecting the correct answer from four options. The activity involves attention, memory, and linguistic skills, while also having a practical application to daily life.<br>**2) Portuguese Popular Sayings** – this task draws upon cultural knowledge and conceptual understanding of popular wisdom. Participants are presented with sentence that is missing a word or expression and must select the correct option from a set of four to complete it in a coherent and meaningful way. This activity trains attentional, linguistic, and long-term memory skills.<br>**3) Household Objects** – in this task, participants pay close attention to a stimulus image and answer a related question by choosing from a set of options. The questions refer to daily living activities and integrate attentional skills, memory, and the linguistic domain.<br>**4) Random** – this task comprises a mixture of the previously described subtypes, presented in a random order.	**c) Screenshot of the Quiz Game: Money**<br>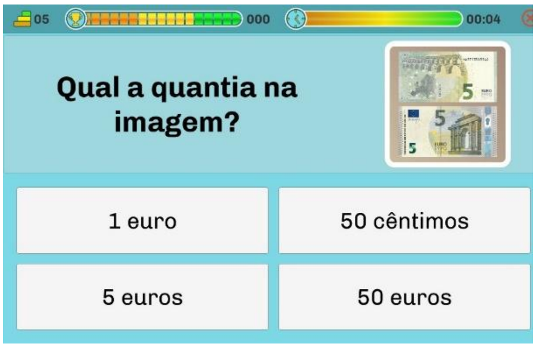

Serious Games description	Illustrative picture
**Reaction Games**<br>This category targets executive functioning, attentional control, and response execution in response to a given stimulus, while inhibiting responses to conflicting instructions. The task involves a stimulus, a circle that expands and contracts, and the participant must follow on-screen instructions (such as "Click when the green circle falls within the bounds of the gray circle.") in order to successfully achieve the objective. The circle´s expansion speed increases after a correct response (*i.e.* when the participant taps the screen at the appropriate moment) and decreases following an incorrect response.	**d) Screenshot of the Reaction Game**<br>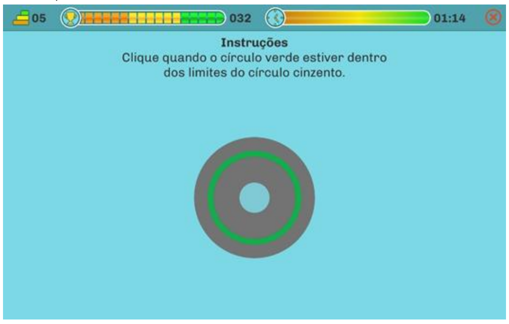
**Whack-a-mole**<br>Whack-a-Mole is a highly interactive game that assesses reaction time to a specific stimulus (moles) and the ability to inhibit responses when a distractor stimulus (flowers) appears. The participant´s task is to tap the screen when a mole emerges and refrain from tapping when a flower appears, thereby "avoiding spoiling the flowers". The game predominantly involves executive functions, attention and concentration, and reactive and inhibitory control in distinguishing between different stimuli.	**e) Screenshot of the Reaction Game: Whack-a-mole**<br>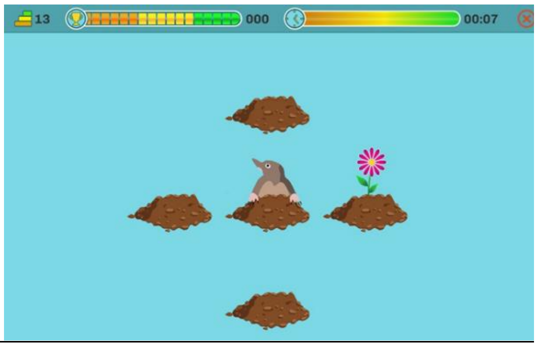
**Pairs**<br>This task is a widely used game for training memory skills. It consists of briefly displaying a set of cards placed face-up in a random arrangement, which are then turned face-down after a few seconds. The task is to use previously processed information and memory strategies to match pairs of cards by turning them over and remembering their positions. The game involves accessing previously stored information and trial-an-error learning, as well as working memory. The game concludes once all pairs have been successfully identified. There are several thematic categories available: Animals, Flowers, Fruit, Architecture, Profession, Tourism, Parties, Sewing, Vehicles, Money and Random, reflecting aspects of daily life and designed to maximize engagement and acceptance by elderly participants.<br><br>**Alternative Pairs**<br>Alternative Pairs is a variation of the previous game that also assesses linguistic and semantic association skills, as well as visuospatial memory. In this version, only one card displays an image, while its matching counterpart shows the corresponding word, a written representation of the image. The task is the same, to match pairs of related cards, but this version adds an additional layer of semantic processing to the memory challenge.	**f) Screenshot of the Pairs Game**<br>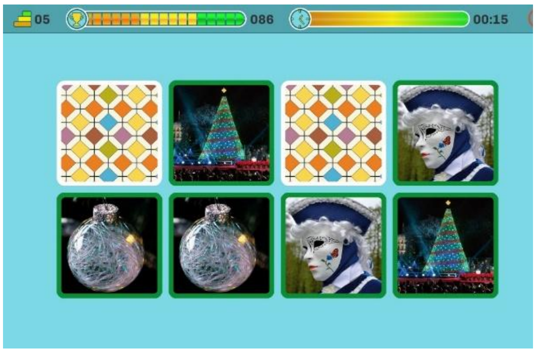<br><br>**g) Screenshot of the Alternative Pairs Game**<br>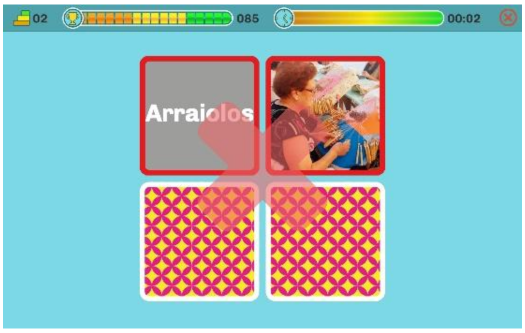

Serious Games description	Illustrative picture

Word, Symbol and Number Search

This game is designed to train executive functions, memory, language and attention. It consists of a set of stimuli, numbers, symbols, words or letters, presented in a grid format. The tasks typically start by briefly displaying a stimulus in isolation (in some cases, it remains visible until the task concludes). The structure and goals of the tasks are divided into four cognitive domains, as follows:

1) Memory

Identify all stimuli in the grid that match a stimulus previously presented in isolation, whether a symbol, letter, word, or image, while ignoring a set of visually similar distractors. If the stimulus is presented as an image, the task may require locating the corresponding word in the grid.

2) Language

From a set of letters, the participant must select a synonym or antonym of a stimulus word. At a more advanced level, the antonym to be identified may appear in reversed form in the grid (for example, “Find the synonym for the following word in reverse”. “COLOCAR”; the answer might be “ROP”).

3) Attention

Search for a stimulus, whether a number, letter, word, symbol, image, or sequence, amongst a set of distractors. The task may require identifying the stimulus in reverse order or matching it to an image, adding an additional layer of difficulty.

4) Executive Functions

From a set of grouped digits, the participant must determine the largest or smallest number that can be formed by rearranging them. The task then involves locating this number in a grid filled with randomly distributed numbers (for example, from “2 1 3” the greatest number is “ 321 ” and the smallest is “ 123 ”).

h) Screenshot of the Word, Symbol and Number Search Game: Memory

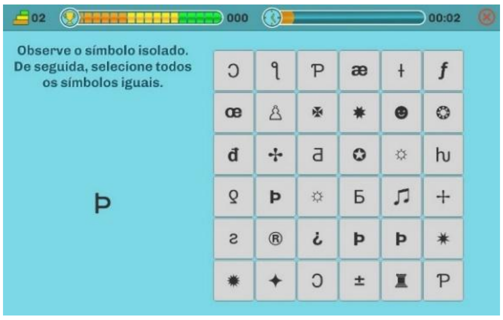

i) Screenshot of the Word, Symbol and Number Search Game: Language

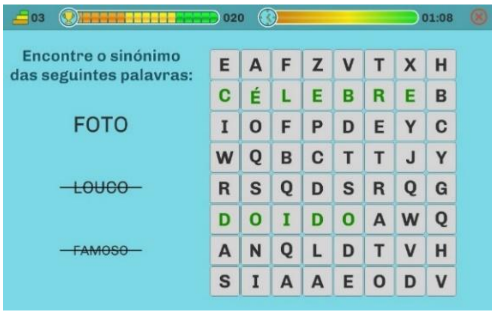

j) Screenshot of the Word, Symbol and Number Search Game: Attention

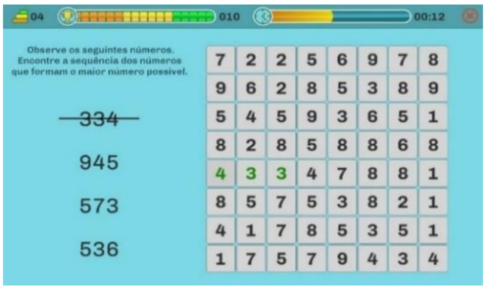

k) Screenshot of the Word, Symbol and Number Search Game: Executive Functions

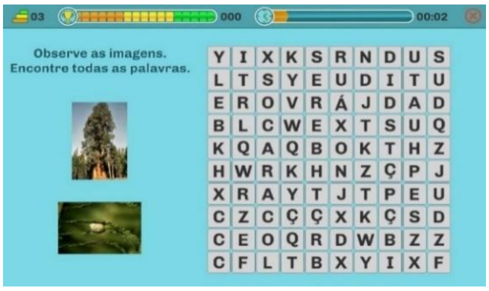

Supplement: sj-pdf-1-dhj-10.1177_20552076261417771 - Supplemental material for Remote cognitive training for older adults using tablets: A pilot trial [file sj-pdf-1-dhj-10.1177_20552076261417771.pdf]
